# Inhibitors of neutrophil recruitment identified using transgenic zebrafish to screen a natural product library

**DOI:** 10.1242/dmm.012047

**Published:** 2013-11-28

**Authors:** Xingang Wang, Anne L. Robertson, Jingyu Li, Ruth Jinfen Chai, Wang Haishan, Pranvera Sadiku, Nikolay V. Ogryzko, Martin Everett, Kanagasundaram Yoganathan, Hongbo Robert Luo, Stephen A. Renshaw, Philip W. Ingham

**Affiliations:** 1 Institute of Molecular and Cell Biology, 61 Biopolis Drive, Proteos, 138673 Singapore.; 2 High-Throughput Molecular Drug Discovery Centre, Tianjin International Joint Academy of Biotechnology and Medicine, Tianjin, 300457 China.; 3 MRC Centre for Developmental and Biomedical Genetics, University of Sheffield, Firth Court, Western Bank, Sheffield, S10 2TN, UK.; 4 Department of Infection and Immunity, University of Sheffield, Sheffield, S10 2JF, UK.; 5 Department of Pathology, Dana-Farber/Harvard Cancer Center, Harvard Medical School, Boston 02215, MA, USA.; 6 Department of Genetics and Cell Biology, Tianjin Key Laboratory of Protein Science, College of Life Sciences, Nankai University, Tianjin, 300071 China.; 7 MerLion Pharmaceuticals Pte Ltd, Science Park II, 117610 Singapore.; 8 Department of Biological Sciences, National University of Singapore, 117543 Singapore.

**Keywords:** Neutrophil, Recruitment, Migration, Drug screen, Zebrafish

## Abstract

Cell migration is fundamental to the inflammatory response, but uncontrolled cell migration and excess recruitment of neutrophils and other leukocytes can cause damage to the tissue. Here we describe the use of an *in vivo* model – the *Tg(mpx:GFP)^i114^* zebrafish line, in which neutrophils are labelled by green fluorescent protein (GFP) – to screen a natural product library for compounds that can affect neutrophil migratory behaviour. Among 1040 fungal extracts screened, two were found to inhibit neutrophil migration completely. Subfractionation of these extracts identified sterigmatocystin and antibiotic PF1052 as the active components. Using the EZ-TAXIScan chemotaxis assay, both compounds were also found to have a dosage-dependent inhibitory effect on murine neutrophil migration. Furthermore, neutrophils treated with PF1052 failed to form pseudopods and appeared round in shape, suggesting a defect in PI3-kinase (PI3K) signalling. We generated a transgenic neutrophil-specific PtdIns(3,4,5)*P*_3_ (PIP3) reporter zebrafish line, which revealed that PF1052 does not affect the activation of PI3K at the plasma membrane. In human neutrophils, PF1052 neither induced apoptosis nor blocked AKT phosphorylation. In conclusion, we have identified an antibiotic from a natural product library with potent anti-inflammatory properties, and have established the utility of the *mpx:GFP* transgenic zebrafish for high-throughput *in vivo* screens for novel inhibitors of neutrophil migration.

## INTRODUCTION

Neutrophils constitute about 40–60% of circulating white blood cells in the human body and are the first line of cellular defence against foreign pathogens deployed by the innate immune system. Neutrophils migrate rapidly to sites of infection or injury in response to diverse chemo-attractants, including N-formyl-methionine-leucine-phenylalanine (fMLF), interleukin-8 and reactive oxygen species (Clark and Klebanoff, 1979; Ellett et al., 2011; Hattori et al., 2010; Lekstrom-Himes et al., 2005). As the insult is contained, the inflammatory response is resolved through reverse migration of neutrophils (Mathias et al., 2006) or by their apoptosis followed by macrophage engulfment (Bratton and Henson, 2011). Uncontrolled neutrophilic activity and continued recruitment of neutrophils to inflammatory sites can result in persistent inflammation, a feature of many human diseases, including chronic obstructive pulmonary disease (COPD) and cystic fibrosis (reviewed by Gernez et al., 2010). Inhaled bronchodilators and corticosteroids are prescribed for COPD, largely for symptom control, but these drugs are, for the most part, ineffective in controlling inflammation and halting disease progression (Morjaria et al., 2010). There is thus a pressing need for novel drugs that specifically target neutrophils.

The zebrafish has emerged as a powerful model not only for the study of vertebrate development but also for high-throughput drug discovery, facilitated by their small transparent embryos that can readily be arrayed into 96-well plates. The generation of transgenic zebrafish expressing neutrophil-specific GFP reporters has made possible the *in vivo* analysis of neutrophil behaviour (Renshaw et al., 2006; Mathias et al., 2006). Like their human counterparts, zebrafish neutrophils migrate towards the wound site in a PI3K-signalling-dependent manner (Yoo et al., 2010) and clear microbes by phagocytosis (Colucci-Guyon et al., 2011). The removal of neutrophils by reverse migration as well as by apoptosis and macrophage uptake has also been observed during inflammation resolution (Ellett et al., 2011; Loynes et al., 2010).

Here we describe the use of one such transgenic zebrafish neutrophil-specific reporter line in an *in vivo* screen of natural product extracts for inhibitors of neutrophil recruitment. We report the identification of two inhibitory compounds using this zebrafish model and their validation using an established *in vitro* mammalian neutrophil migration assay.

## RESULTS

### Tailfin resection-induced migration provides a robust *in vivo* screening assay

**Fig. 1.**
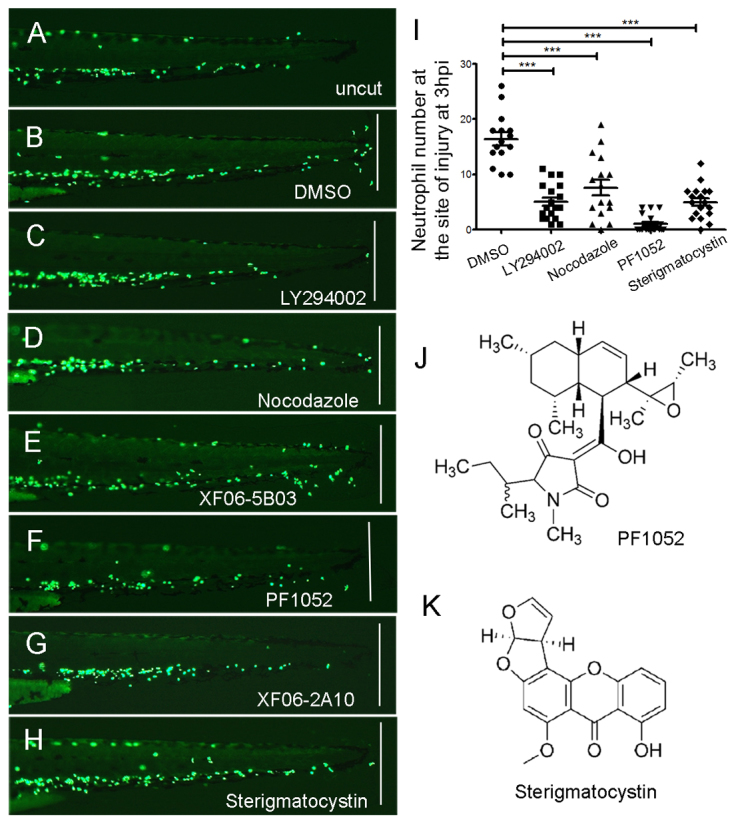
**Inhibitory effect of PF1052 and sterigmatocystin on neutrophil migration towards the wound site.** Neutrophil migration assay was carried out using *Tg(mpx:GFP)^i114^* larvae according to Renshaw et al. (Renshaw et al., 2006). (A) Neutrophils were quiescent on an uninjured 3-dpf larva. (B) About ten neutrophils were recruited to the wound site 3 hours after the tailfin was amputated on a control larva that was treated with DMSO. (C,D) Larvae treated by PI3K inhibitor LY294002 (50 μM) or microtubule inhibitor nocodazole (33 μM), respectively, had few neutrophils recruited to the wound. (E) An extract (ID: XF06-5B03) from an ascomycete genus *Sphaeropsidales* completely blocked neutrophil migration towards the wound at 50 μg/ml. (F) The active component identified from XF06-5B03 extract (PF1052) also completely blocked neutrophils recruitment at a very low concentration of 2 μM; the molecular structure of PF1052 is shown in J. (G,H,K) Another extract, XF06-2A10, from a fungus, genus *Aspergillus*, that blocked the migration of neutrophils contains the functional component sterigmatocystin, working at 50 μM. (I) Quantification of neutrophils recruited to the site of injury (*n*>15). The white line on B-H indicates the amputation site of tailfin. ***P<0.001.

Our aim was to establish an assay for the rapid identification of highly effective inhibitors of neutrophil migration. To this end, we constructed an assay protocol based on rapid visual assessment of neutrophil recruitment. Targeted expression of GFP, using the myeloperoxidase (*mpx*) or lysozyme C (*lyz*) promoters (Renshaw et al., 2006; Hall et al., 2007), reveals the anterior yolk sac and posterior intermediate cell mass (ICM) of living zebrafish larvae to be the origin of neutrophils. In uninjured larvae, caudal neutrophils remain along the ventral side (Fig. 1A) but, following tailfin amputation, they rapidly migrate towards the wound site. By 3 hours after amputation, ~ten neutrophils are recruited to the distal portion of the remaining tailfin (Fig. 1B). To determine whether this assay could be used to screen for compounds that affect neutrophilic migration, rather than those that accelerate inflammation resolution (Ellett et al., 2011; Loynes et al., 2010), 2- to 3-day post-fertilisation (dpf) larvae were pre-incubated with the known PI3K inhibitor LY294002 and microtubule inhibitor nocodazole for 1, 3 or 5 hours prior to tailfin transection, and subsequently transferred into fresh drug-containing media for a further 3 hours before analysis of neutrophil recruitment to the wound site (see Materials and Methods). We found that both LY294002 (50 μM) and nocodazole (33 μM) were effective in inhibiting neutrophil migration (Fig. 1C,D,I) after 3 or 5 hours of pre-incubation. Because there were more toxic effects following 5 hours of pre-incubation, we used a 3-hour pre-incubation for the screening protocol.

**TRANSLATIONAL IMPACT**

**Clinical issue**

Neutrophil-mediated inflammation is a feature of a diverse range of diseases, including chronic obstructive pulmonary disease (COPD), kidney disease, inflammatory bowel disease and neutrophilic dermatoses (a group of skin disorders). Collectively, these diseases are a major threat to global health: COPD alone is currently the fifth biggest killer globally and is predicted by the WHO to rise to the third position by 2020. This is exacerbated by the complete lack of compounds that target the neutrophil in clinical practice, highlighting an area of huge unmet need. Existing approaches to drug discovery rely on identification of targets, drugging those targets using compounds from pharmaceutical libraries, and then determining the cellular phenotype. By contrast, a phenotype-based screen is utilised here to identify compounds with anti-inflammatory actions from natural compound libraries.

**Results**

This report describes the development of a rapid assay for the identification of compounds with activity against neutrophil recruitment following tissue injury in zebrafish expressing GFP-labelled neutrophils. Using this assay on a carefully chosen library of fungi-derived natural products, followed by fractionation and mass spectrometry, two compounds, including one antibiotic, were identified with profound effects on neutrophil recruitment *in vivo*. Both compounds were also found to have inhibitory effects on murine neutrophil migration *in vitro*. Using a newly generated transgenic zebrafish line, differences in activity between antibiotic PF1052 and inhibitors of phosphoinositide 3-kinases (a family of enzymes with multiple cellular roles) were identified, suggesting that PF1052 acts independently of these signalling pathways.

**Implications and future directions**

These findings demonstrate that zebrafish can be used for robust *in vivo* assessments of compound activity at a level of throughput that facilitates drug discovery. Furthermore, this study shows that pure and highly active compounds can be identified from natural product extracts and their mechanism of action can be explored *in vivo*. Finally, the compounds identified might have value for elucidating the mechanisms of neutrophil chemotaxis in the context of disease, and represent viable candidates to lead future programmes of anti-inflammatory drug discovery.

### A screen of fungal extracts uncovers naturally occurring neutrophil inhibitors

Using the tailfin transection assay, we screened a library of 1040 crude fungal extracts from the MerLion collection, a diverse assortment of natural products obtained from a variety of sources. From this library, we identified 35 candidate samples that met our predetermined criteria and further validation of these 35 samples confirmed that two of them produce consistent inhibitory effects (see supplementary material Table S1). The first of these, XF06-5B03, completely inhibited all neutrophil migration to the wound site (Fig. 1E). This extract was derived from an ascomycete of the genus *Sphaeropsidales*, isolated from a soil sample collected in Singapore. The second extract, XF06-2A10, derived from the *Aspergillus* genus, similarly inhibited neutrophil recruitment (Fig. 1G).

### Identification of antibiotic PF1052 and sterigmatocystin as neutrophil migration inhibitors

A major challenge posed by screening a natural product library is the identification of the active component in the biological mixture. To identify the active component(s) identified by the tailfin assay, each extract was fractionated by high performance liquid chromatography (HPLC) into 38 fractions. Remarkably, only a single fraction of XF06-5B03 produced the same response as the crude extract. Liquid-chromatography–mass-spectrometry (LC-MS) analysis of the active fraction identified a chemical structure corresponding to a compound within the MerLion Pharmaceuticals purified natural product compound library. This compound, termed antibiotic PF1052 (CAS No. 147317-15-5), is a tetramic acid (Fig. 1J) first described by Meiji Seika Kaisha, Ltd as having antimicrobial properties (Sasaki et al., 1992). A small amount of the pure compound was obtained for further evaluation and confirmed as being the active component within the fraction. We also purchased PF1052 from an independent source (Enzo Life Sciences) and verified its activity as a highly effective neutrophil migration inhibitor, with an effective concentration as low as 2 μM (Fig. 1F,I).

Fractionation and LC-MS analysis of the second extract, XF06-2A10, revealed its active component to be sterigmatocystin (Fig. 1L). Pure sterigmatocystin powder purchased from Sigma-Aldrich reproduced the effect of XF06-2A10 at 50 μM, confirming its identity as the active component (Fig. 1H,I).

PF1052 works as antibiotic on bacteria *in vitro* at 2.3 mM (Koyama et al., 2005). We tested whether this concentration was comparable to that within the embryos by performing LC-MS analysis of zebrafish larvae pre-treated for 3 hours. Using LC-MS analysis, we found that absorption of PF1502 by zebrafish larvae was rapid, the concentration reaching 219 μM by 3 hours and 301 μM by 6 hours, with a concomitant fall in media concentration from 2 μM to 0.67 and 0.44 μM, respectively (Table 1). Larvae incubated in 50 μM sterigmatocystin had concentrations of 870 and 704 μM by 3 and 6 hours, respectively. The concentration of PF1052 is much lower than the published effective antibiotic concentration, suggesting a different mechanism for its neutrophil migration inhibitory effects.

**Table 1:**
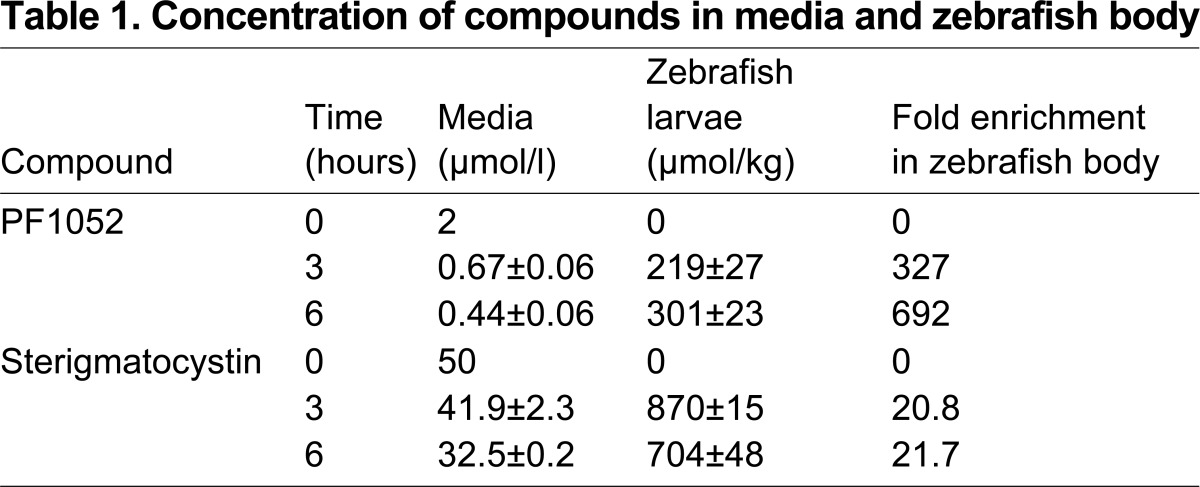
Concentration of compounds in media and zebrafish body

### Effects of PF1052 and sterigmatocystin on mammalian neutrophil migration

**Fig. 2.**
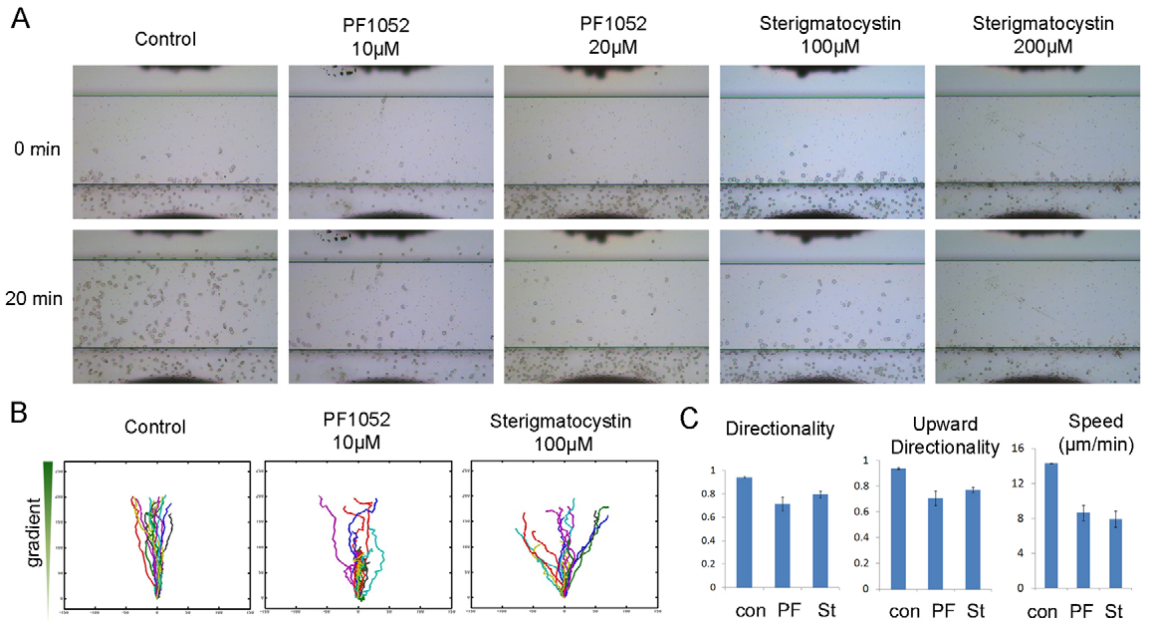
**PF1052 and sterigmatocystin reduced mouse neutrophil chemotaxis.** Mouse neutrophils were pre-treated with 10 μM or 20 μM PF1052, 100 μM or 200 μM sterigmatocystin, or DMSO as control, then were exposed to a chemoattractant gradient generated by addition of 1 μM fMLF (1 μl) in the EZ-TAXIScan device. (A) Chemotaxis of mouse neutrophils in response to chemoattractant fMLF, both PF1052 (10 μM) and sterigmatocystin (100 μM) severely inhibited neutrophils migrating upwards, compared with DMSO treatment. At higher concentrations (PF1052 20 μM; sterigmatocystin 200 μM), very few neutrophils were migratory. (B) Cell tracks of migrating neutrophils (cells that move at least 65 μm from the bottom of the channel) (*n*=20). (C) Effect of PF1052 and sterigmatocystin treatment on mouse neutrophil chemotaxis. Neutrophils were evaluated for migration speed, directionality and upward directionality. Results show the means (±s.d.) of three independent experiments, *P*<0.05 versus control neutrophils (Student’s *t*-test).

We next asked whether the compounds identified using the zebrafish assay could also inhibit the migration of mammalian neutrophils using an EZ-TAXIScan chemotaxis device, in which a stable chemo-attractant gradient is formed in a 260-μm-wide channel (Fig. 2A). Freshly purified mouse neutrophils (treated with DMSO as control) migrated robustly up the gradient (see supplementary material Movie 1). By contrast, fMLF (a formyl peptide)-induced chemotaxis of neutrophils exposed to 10 μM PF1052 or 100 μM sterigmatocystin was severely compromised, with most neutrophils losing motility and polarity. Although a few cells managed to migrate out, they showed a lack of directionality and slow migration up the chemo-attractant gradient (Fig. 2B,C; also see supplementary material Movies 2 and 3). The effect was concentration dependent, with 20 μM PF1052 and 200 μM sterigmatocystin having a stronger effect (Fig. 2A; also see supplementary material Movies 4 and 5).

### PF1052 is a specific inhibitor of neutrophil migration

**Fig. 3.**
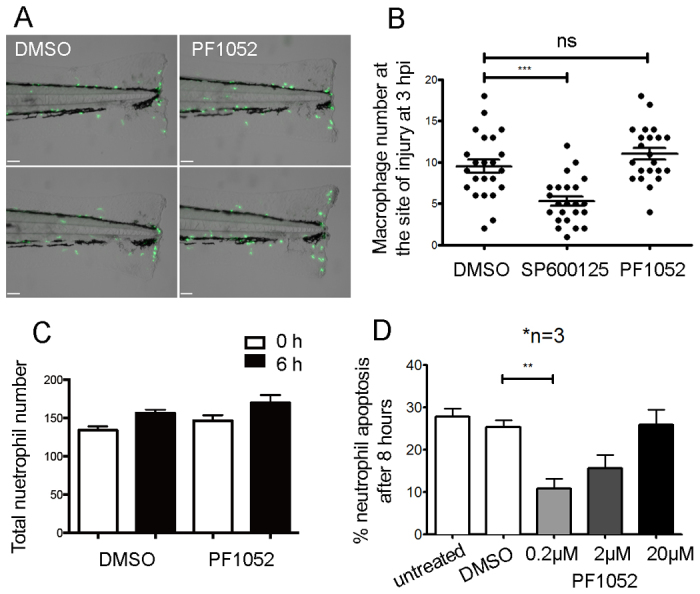
**PF1052 is a specific inhibitor of neutrophil migration.** (A,B) PF1052 has no significant effect on macrophage migration; the MAP kinase inhibitor SP600125 was used at 30 μM as a positive control. (C) PF1052 did not significantly reduce total neutrophil number in intact larvae. (D) PF1052 does not induce human neutrophil apoptosis and seems to promote survival at a low concentration (**P<0.01; ***P<0.001; ns, not significant).

Because sterigmatocystin is both hepatotoxic and carcinogenic, we excluded it from further analysis and focused exclusively on PF1052. To investigate whether the effect of PF1052 on migration is specific to neutrophils, we monitored macrophage migration to a tailfin wound in PF1052-treated larvae and found no difference between treated and control animals (Fig. 3A,B). This is consistent with the inhibitory activity of PF1052 being specific to neutrophils rather than a general effect on innate immune cell migration.

To confirm that PF1052 acts to block the recruitment rather than the ontogeny of neutrophils, whole-body neutrophil counts were performed in the presence of the active compound or vehicle-only control. No differences in neutrophil number were observed following PF1052 treatment and the expected developmental increase in neutrophil number still occurred in the presence of PF1052 (Fig. 3C). We also tested the effect of PF1052 on the viability of human neutrophils *in vitro*. Human neutrophils cultured with or without a range of doses of PF1052 were assessed morphologically for apoptosis. At lower concentrations (200 nM and 2 μM) PF1052 suppressed the apoptosis of isolated human neutrophils; at higher concentrations, however, this suppressive effect was lost (Fig. 3D).

### PF1052 inhibits neutrophil migration and affects pseudopodia formation

**Fig. 4.**
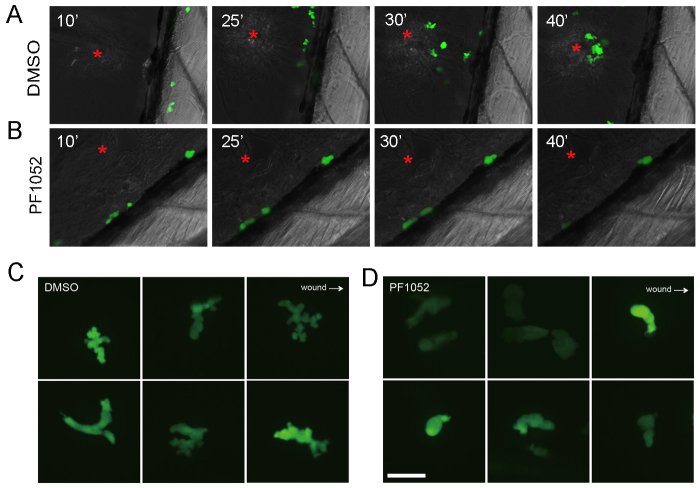
**Time-lapse imaging of neutrophil migration *in vivo*.** The ventral fin of 6-dpf larvae was injured by a sharp syringe needle, and chemotaxis of neutrophils were imaged by spinning disk microscope. (A) In a control larva treated with DMSO, neutrophil migration was not directed until 10 minutes after fin injury. Thereafter, they migrated actively towards the wound site (red asterisk; 25 and 30 minutes); by 40 minutes after injury, around eight neutrophils were recruited. (B) In contrast to the control, neutrophils treated with PF1052 remained stationary during 40 minutes of imaging; they appeared round and barely formed any pseudopods. (C) Higher magnification shows that DMSO-treated neutrophils actively formed multiple pseudopods and this was impaired in PF1052 treated-neutrophils (D).

Using time-lapse imaging, we investigated how PF1052 blocks neutrophil migration to the wound site. In control larvae, treated only with DMSO, neutrophils began migrating towards the wound 10 minutes after ventral fin resection. During migration, the cells actively extended pseudopodia to sense the direction of the wound site (Fig. 4A,C). By 40 minutes, ~eight neutrophils had been recruited to the site of injury (red star in Fig. 4A). By contrast, no neutrophils were recruited to the wound site in PF1052-treated embryos. They appeared static during the 40 minutes of imaging, remaining rounded and barely able to form any pseudopodia (Fig. 4B,D).

### PF1052 acts independently of PI3K and AKT activation

**Fig. 5.**
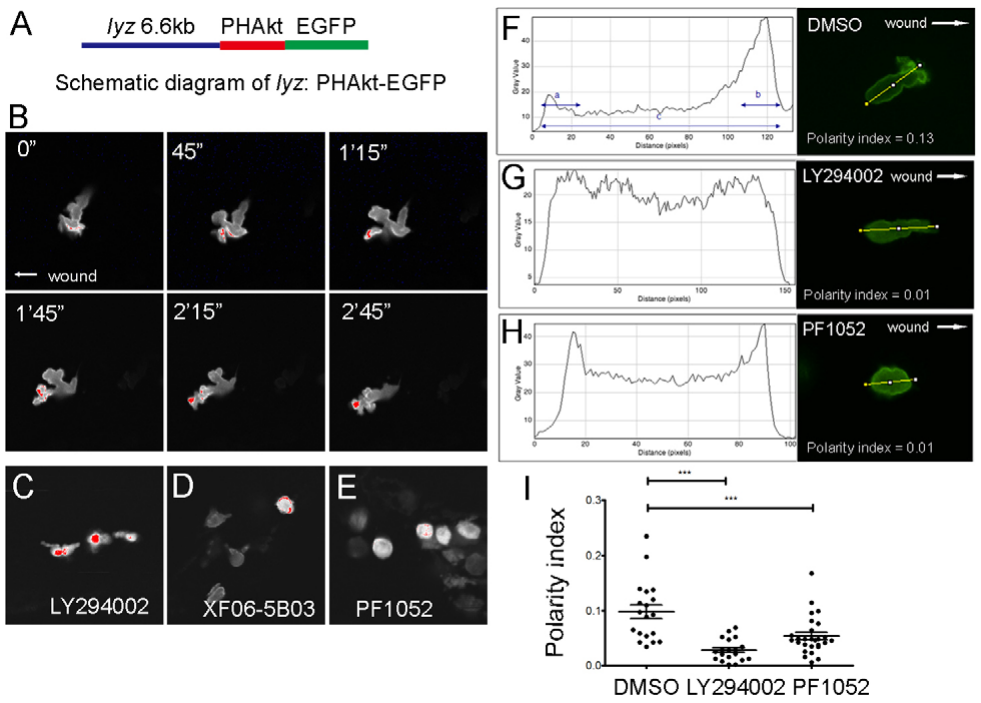
**PF1052 inhibited neutrophil migration in a PI3K-independent manner.** (A) Schematic diagram of the *lyz:PHAkt-EGFP* construct used to generate the stable transgenic line. (B) Time-lapse imaging of migrating neutrophils showed PHAkt-EGFP signal was always localised to the cell front in control larvae. The red signal indicates the highest level of PI3K at the leading edge. (C) PI3K inhibitor LY294002 reversed the cellular localisation of PI3K, which accumulated within the cell body. (D,E) Extracts from *Sphaeropsidales* and its functional component PF1052 did not affect the localisation of PI3K at the cell membrane. (F-I) Quantification of neutrophil polarity index in DMSO-, LY294002- and PF1052-treated larvae. LY294002 and PF1052 significantly reduced neutrophil polarity index (***P<0.001).

The role of PI3K signalling in regulating F-actin polymerisation to promote neutrophil migration is well established. During migration, PI3K is localised to the leading edge of neutrophils, catalysing the phosphorylation of PtdIns(4,5)*P*_2_ (PIP2) to generate PtdIns(3,4,5)*P*_3_ (PIP3), which binds the PH domain of AKT to activate F-actin polymerisation at the cell front. To investigate whether PF1052 inhibits PI3K directly, we generated a transgenic line expressing an *in vivo* sensor of PIP3 (see Materials and Methods) to probe PI3K subcellular localisation (Fig. 5A). In *Tg(lyz:PHAkt-EGFP)^i277^* larvae, fluorescent signal accumulated at the leading edge of neutrophils, usually in the pseudopodia (Fig. 5B). When two pseudopodia extended from a single neutrophil during chemotaxis, the one with higher levels of PHAkt-EGFP signal predicted the direction of migration. In *Tg(lyz:PHAkt-EGFP)* larvae treated with the PI3K inhibitor LY294002, accumulation of fluorescent signal was lost at the leading edge, but instead was dispersed throughout the cell body (Fig. 5C). By contrast, in larvae treated with XF06-B03 or PF1052, EGFP signal accumulated at the periphery of the neutrophils, despite their loss of polarity (Fig. 5D,E).

For a more robust quantification of the effect of PF1052 on neutrophil polarity, we devised a measurement termed the ‘polarity index’ that reflects the difference in EGFP intensity at the leading edge of the cell compared with the trailing edge, as well as the total fluorescence within the cell. 3-dpf *Tg(lyz:PHAkt-EGFP)^i277^* larvae were pre-incubated with either 2 μM PF1052, 50 μM LY294002 or DMSO control for 3 hours, followed by tailfin transection. Individual neutrophils were imaged and the fluorescence intensity was quantified along a longitudinal transection of each cell to calculate the polarity index (as described in Materials and Methods). The majority of neutrophils from DMSO-control-treated larvae appeared polarised, with the most intense EGFP expression in pseudopodia at their leading edge and only a faint EGFP signal within the cell body (Fig. 5F). Consistent with our previous observations, neutrophils from LY294002- or PF1052-treated larvae did not have a defined leading edge (Fig. 5G,H), which was reflected in their significantly reduced polarity index compared with the DMSO control (Fig. 5I).

**Fig. 6.**
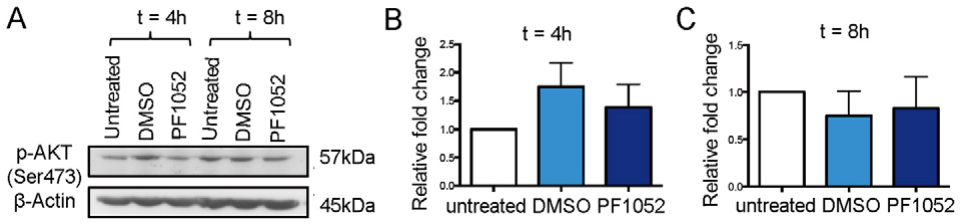
**PF1052 does not inhibit AKT phosphorylation in human neutrophils.** (A) Western blotting shows PF1052 does not affect AKT phosphorylation in human neutrophils after either 4 or 8 hours culture. (B,C) Quantification of the level of p-AKT in untreated, DMSO- and PF1052-treated samples.

The pattern of EGFP signal observed in neutrophils from PF1052-treated larvae indicated that this compound acts independently of PIP3 generation at the cell membrane. We therefore tested whether PF1052 prevented the phosphorylation of AKT by inhibition of activating kinases at the membrane. To investigate this, we examined expression levels of phosphorylated AKT (p-AKT) following PF1052 treatment in human neutrophils using western blotting techniques. We found no change in p-AKT expression in neutrophils following either 4 or 8 hours culture with PF1052 (Fig. 6A–C). These data, along with our *in vivo* observations in the zebrafish, suggest that PF1052 acts independently of the PI3K-Akt signalling pathway to disrupt leading-edge specification and induce its inhibitory effect on neutrophil migration.

## DISCUSSION

We have successfully used a transgenic zebrafish model in what we believe to be the first *in vivo* screen for naturally occurring inhibitors of neutrophil migration. Through subfractionation and LC-MS analysis, we identified the antibiotic PF1052 and sterigmatocystin as the active components of the two fungal extracts showing inhibitory activity. When applied to mouse neutrophils, both compounds identified in the zebrafish screen showed a dose-dependent inhibitory effect on migration, with an inhibition of pseudopod formation similar to that observed in their zebrafish counterparts. These findings further validate the utility of the *Tg(mpx:GFP)^i114^* zebrafish as a model system for the analysis of human neutrophil pathobiology and illustrate the efficacy of such phenotypic assay systems for screening complex natural product extract mixtures.

Because sterigmatocystin is reported to have carcinogenic properties (Ellett et al., 2011), we chose to focus our attention on PF1052, the therapeutic potential of which has not previously been explored. PF1052 was initially isolated from fermentation broth of the fungus Phoma sp. and shown to have antimicrobial activity (Takahashi et al., 1996). It is structurally closely related to spylidone, also isolated from Phoma sp. fermentation broth, but unlike spylidone, PF1052 has no effect on lipid droplet accumulation in macrophages (Koyama et al., 2005). In this study, we found that PF1052 had no inhibitory effect on macrophage migration.

The effect of PF1052 on neutrophils was distinct from that of the PI3K inhibitor LY294002; neutrophils treated with the latter retain their ability to form thin pseudopods and PHAkt-GFP localises to the centre of the cell (Yoo et al., 2010). By contrast, treatment with PF1052 had no effect on Akt phosphorylation or PHAkt-EGFP localisation but caused loss of polarisation and pseudopod formation, reflected in the significantly reduced neutrophil ‘polarity index’. These effects are similar to those caused by a dominant-negative form of human Rac2D57N or morpholino-mediated knockdown of Rac2 (Deng et al., 2011). There are also similarities to the phenotype of mouse neutrophils mutant for SHIP, a Src homology 2 (SH2)-domain-containing inositol-5-phosphatase, which hydrolyses PI(3,4,5)P3 to PI(3,4)P2. SHIP was believed to coordinate with PI3K to determine the localisation of PIP3 and PIP2, thus controlling neutrophil polarity and motility (Nishio et al., 2007). In zebrafish, however, knockdown of SHIP leads to an increase in neutrophil motility and infiltration to the wound, whereas overexpression of SHIP impairs such migration (Lam et al., 2012). Although further analysis is needed to resolve the disparity between these two data sets, we are inclined to argue against an inhibitory effect of PF1052 on SHIP, based on the findings in zebrafish. Additional studies will be required to identify the specific target(s) of PF1052 in neutrophils.

### Summary

Here, we demonstrate a feasible *in vivo* assay for the discovery of compounds with anti-inflammatory actions from natural compound libraries. Testing over 40 compounds with suitable positive and negative controls can easily be accomplished in a day by a single operator. This screen has identified a novel and highly effective neutrophil migration inhibitor, PF1052, with the potential to inform our understanding of neutrophil chemotaxis. We have found that this compound acts independently of the PI3K and AKT enzymes and might either act downstream of these molecules or act via other pathways. Identification of the molecular target has the potential to reveal a novel target pathway for future drug discovery programmes.

## MATERIALS AND METHODS

### Zebrafish husbandry

Adult fish were maintained on a 14-hour light/10-hour dark cycle at 28°C in the AVA certificated IMCB Zebrafish Facility (Singapore). The *Tg(mpx:GFP)^i114^* line (Renshaw et al., 2006) was used for neutrophil assays. To investigate macrophage recruitment, we generated a *Tg(mpeg1:Gal4.VP-16)^sh256^* line as previously described (Ellett et al., 2011) and crossed this to the *Tg(UAS:Kaede)^s1999t^* line (Davison et al., 2007) to generate double-transgenic larvae expressing green fluorescent Kaede protein under the macrophage-specific *mpeg1* promoter.

### Creation of the *Tg(lyz:PHAkt-EGFP)* line

The 6.6 kb of lysozyme C promoter (Hall et al., 2007) was cloned into a gateway vector p5E-MCS (Tol2kit). The PH domain of Akt was amplified from zebrafish cDNA by PCR using forward primer 5′-GGGGACAAGTTTGTACAAAAAAGCAGGCTCCACCATGGGCATGA-ACGAGATCAGCGTCGT-3′ and reverse primer 5′-GGGGACCAC -TTTGTACAAGAAAGCTGGGTCACCCAGCAGCTTTAAGTAGTCAA-3′, and the PCR product was recombined with pDONR 221 (Invitrogen) to produce pME-PHAkt. The final lyz:PHAkt-EGFP construct was created by recombination of p5E-lysC, pME-PHAkt, p3E-EGFP and pDestTol2pA2 (Tol2kit) using LR Clonase II plus (Invitrogen). The DNA construct was co-injected with *Tol2* mRNA into one-cell-stage embryos to produce the transgenic line.

### Reagents

The microtubule inhibitor nocodazole (M1404), PI3K inhibitor LY294002 (L9908), sterigmatocystin (S3255) and Tricaine (E10521) were ordered from Sigma-Aldrich (St Louis, MO). PF1052 (ALX-380-147) was purchased from Enzo Life Sciences (Farmingdale, NY).

### Natural product library

Crude fungal extracts were prepared by fermenting fungal strains in a variety of growth media and extracting the freeze-dried products of fermentation using methanol, followed by removal of solvent by rotary and centrifugal evaporation. 1 mg of each dried crude extract was dispensed per well of a 96-well microtitre plate.

### Extract fractionation

The fungal extract was prepared at a concentration of 10 mg/ml in DMSO and centrifuged at 15,000 ***g*** for 2 minutes. An aliquot of extract (up to 800 μg or 80 μl) was analysed by HPLC [Thermos Hypersil BDS C18 4.6×150 mm, 5 μm, solvents: (A) H_2_O + 0.1% formic acid, (B) MeCN + 0.1% formic acid; gradient: 0 minutes 0% B, 22 minutes; 100% B, 32 minutes; 100% B; flow: 1 ml/minute; 30°C). The eluent from the DAD (Agilent 1100 with UV detection at 210, 254, 354 and 480 nm) was split in a 1:10 ratio between an Esquire 3000 ion trap mass spectrometer (Bruker Daltonik GmbH, Bremen, Germany) and a fraction collector configured to collect into a 96-well microtitre plate (0.79 minutes/well). A total of 38 wells were collected (0-30 minutes). The 96-well microtitre plate was dried using the Genevac HT-8 centrifugal evaporator (8-hour cycle) at 40°C. Wells 39 and 40 were used as controls, which contained 125 μg and 250 μg of crude extract, respectively. After complete evaporation of the solvent, materials in the wells were analysed for activity against the tailfin resection assay as described below. Active fractions were analysed by MS and MS-MS, and data matched against MerLion’s Esquire compound library, containing mass spectra records of 2496 compounds that have been analysed under the same conditions.

### Measurement of compound concentration by LC-MS

Zebrafish larvae treated with test compound were mixed with glass beads and 0.1 M of KH_2_PO_4_ buffer (150 μl), and smashed with TOMY Micro smash MS-100 (4500 rpm, 30 seconds ×4). After adding acetonitrile (350 μl), the mixture was shaken for 30 seconds ×2, and centrifuged at 20,000 ***g***, 4°C, for 10 minutes for two cycles. Supernatant was separated for LC-MS analysis, which was carried out on a Waters 2795 Separations Module equipped with a Waters 2996 Photodiode Array (PDA) detector and micromass Quattro micro mass spectrometer.

### Zebrafish *in vivo* cell migration assays

*Tg(mpx:GFP)^i114^* transgenic zebrafish larvae (2-3 dpf) were first exposed to extracts in a 96-well plate format, with three larvae per well, containing 200 μl of fish water, 1 mM of Tris pH 7.4, 1% DMSO and 10 μg fungus extract, at 28°C. Three hours later, the larvae were anaesthetised with Tricaine and their tailfins amputated using sharp needles to induce the migration of neutrophils. After injury, the zebrafish larvae were transferred to fresh extract solution for 3 hours before visual inspection using a fluorescence stereomicroscope. The number of neutrophils recruited to the wound site was counted. The effect of PF1052 on macrophage migration was assessed in the same manner, using the *Tg(mpeg:Gal4;UAS:Kaede)^sh256^* line.

### Mouse neutrophil chemotaxis assay

The EZ-TAXIScan chamber (Effector Cell Institute, Tokyo, Japan) was assembled with a 260 μm wide × 4 μm thick silicon chip on a 2 mm untreated glass base, as described by the manufacturer, and filled with HBSS (with Ca^2+^ and Mg^2+^)/0.2% BSA. Murine neutrophils were isolated from bone marrows of 8-week-old male C57BL/6 mice (the Jackson Laboratories, Bar Harbor, ME). Drug-treated (or DMSO-treated) murine neutrophils (1 μl, 10×10^6^/ml) were added to the lower reservoir of each of the six channels and allowed to line up by removing 18 μl of buffer from the upper reservoir. HBSS (with Ca^2+^ and Mg^2+^)/0.2% BSA (15 μl with the appropriate pharmacological inhibitor) was then added to fill both reservoirs, which were maintained at 37°C. 1 μl of 1 μM chemoattractant (fMLF) was then added to the upper reservoir and neutrophil migration in each of the channels was captured sequentially every 30 seconds for 20 minutes using a 10× lens on a Discovery Screening System (Universal Imaging Corporation, Downingtown, PA). Drug PF1052 or sterigmatocystin was added directly to murine neutrophils (100 μl, 10×10^6^/ml) in HBSS (with Ca^2+^ and Mg^2+^)/0.2% BSA and incubated in a 37°C, 5% CO_2_ chamber for 30 minutes before the chemotaxis assay.

### Analysis of cell tracks and morphology

The (*x*,*y*) coordinates of migrating neutrophils (i.e. neutrophils that cross >65 μm from the starting line) were tracked from sequential images using DIAS imaging software (Solltech, Oakdale, IA). Cell tracks were then realigned such that all the cells started from the same starting point (0,0) and plotted using Matlab (MathWorks, Natick, MA). Directionality (0 to 1) is defined as straight-line migration distance from the origin divided by the total migration length. Upward directionality (−1 to 1) is defined as straight-line distance migrated in the upward direction divided by total migration length. Migration speed (μm/minute) was calculated as the average of cell speeds (migration distance between the current frame and the previous frame divided by the time between sequential frames, 0.5 minutes) at each captured frame.

### *In vitro* apoptosis assay

Neutrophils were isolated from healthy volunteers as previously described (Wardle et al., 2011), with approval of the South Sheffield Research Ethics Committee (reference number: STH13927). Neutrophils were cultured with DMSO, or 0.2, 2 or 20 μM PF1052 for 8 hours at 37°C, and apoptosis was assessed by morphology on cytospins stained with Quick-Diff (Gentaur, Brussels, Belgium).

### Detection of p-AKT by western blot analysis

Neutrophils were isolated and cultured as described above, for either 4 or 8 hours, with 2×10^6^ cells per condition. Cells were lysed as previously described (Wardle et al., 2011) and western blotting was performed according to standard protocols (Sambrook, 1989). Antibodies used were anti-phospho-Akt (Ser473) (Cell Signaling, Herts, UK) at 1:1000, or anti-β-actin (Sigma-Aldrich, Poole, UK) at 1:4000, with polyclonal goat anti-rabbit secondary antibody (Dako, UK) at 1:2000. To analyse changes in protein expression, densitometry was performed using ImageJ, as previously described (Wardle et al., 2011).

### Imaging of zebrafish neutrophil migration *in vivo*

For time-lapse imaging, *Tg(lyz:PHAkt-EGFP)^i277^* zebrafish larvae were anaesthetised with 0.02% Tricaine and embedded in 1% low melting agarose on a depression slide. Embedded larvae were then placed under a 40× water objective, immersed in water containing Tricaine. Images were captured every 15 seconds, using an Olympus spinning disc fluorescence microscope (BX61) or Olympus Confocal microscope (BX61). To assess the effects of PF1052 on neutrophil polarity, *Tg(lyz:PHAkt-EGFP)^i277^* larvae were pre-incubated with either 2 μM PF1052, 50 μM LY294002 or DMSO control for 2 hours prior to tailfin transection and mounting. Images of individual neutrophils in the region between the injury site and the posterior blood island were captured using an UltraVIEWVoX spinning disk confocal imaging system (PerkinElmer Life and Analytical Sciences) with an inverted Olympus IX81 microscope, at 60× magnification with ten Z slices. Images were analysed in ImageJ, by drawing a transection through each neutrophil from the trailing edge towards the leading edge. In cases where there were no clear leading or trailing edges, the line was drawn through the most longitudinal section of the cell. A plot profile was generated to measure the fluorescence intensity per pixel along the length of the line and the mean intensities along sections of this line (as defined in Fig. 5A) were used to calculate the polarity index, with the equation:

polarity index=|log10ab|×a+bc.

### Statistical analysis

Data were analysed in GraphPad Prism 5.0 using one-way ANOVA with appropriate post-test adjustment.

## Supplementary Material

Supplementary Material
