# Supplementary material for: Inhibitors of neutrophil recruitment identified using transgenic zebrafish to screen a natural product library
Source: Dis Model Mech. 2013 Nov 28;7(1):163–9. doi: 10.1242/dmm.012047 (PMC3882058; doi:10.1242/dmm.012047)
Supplement: Supplementary Material [file supp_7_1_163__index.html]

Inhibitors of neutrophil recruitment identified using transgenic zebrafish to screen a natural product library — Supplementary Material 

# Inhibitors of neutrophil recruitment identified using transgenic zebrafish to screen a natural product library

## DMM012047 Supplementary Material

**Files in this Data Supplement:**

- **Supplementary Material PDF**
