# Supplementary material for: Inhibitors of neutrophil recruitment identified using transgenic zebrafish to screen a natural product library
Source: Dis Model Mech. 2013 Nov 28;7(1):163–9. doi: 10.1242/dmm.012047 (PMC3882058; doi:10.1242/dmm.012047)
Supplement: Supplementary Material [file supp_012047_DMM012047.pdf]

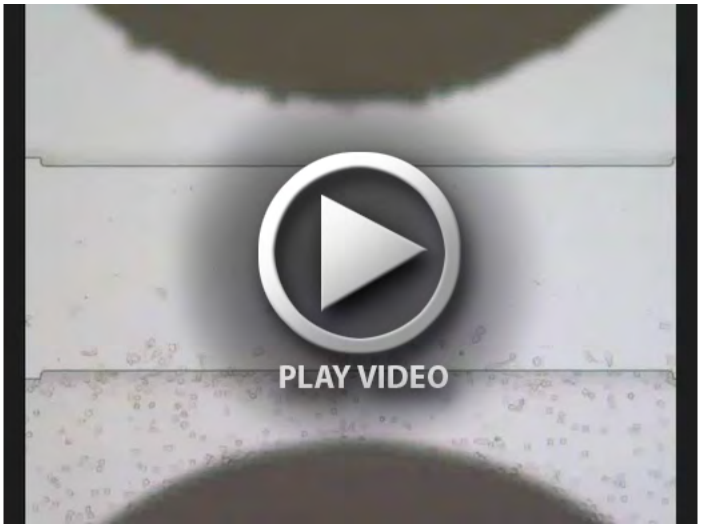

Movie S1. fMLF-induced mouse neutrophil chemotaxis treated by DMSO.

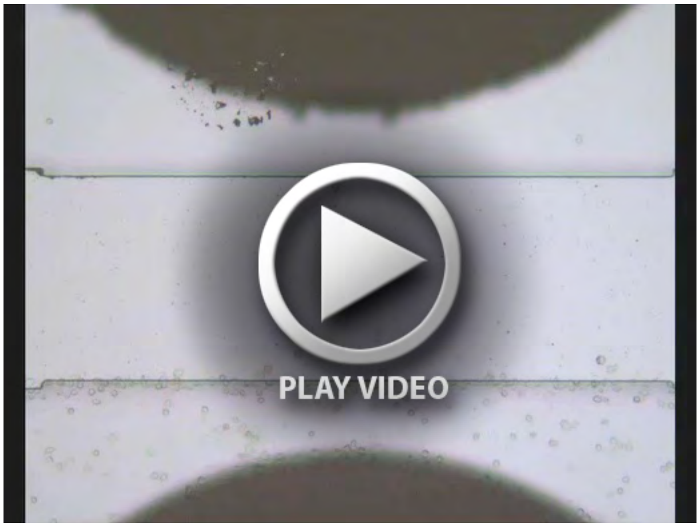

Movie S2. Inhibitory effects of PF1052 on mouse neutrophil chemotaxis at 10 μM.

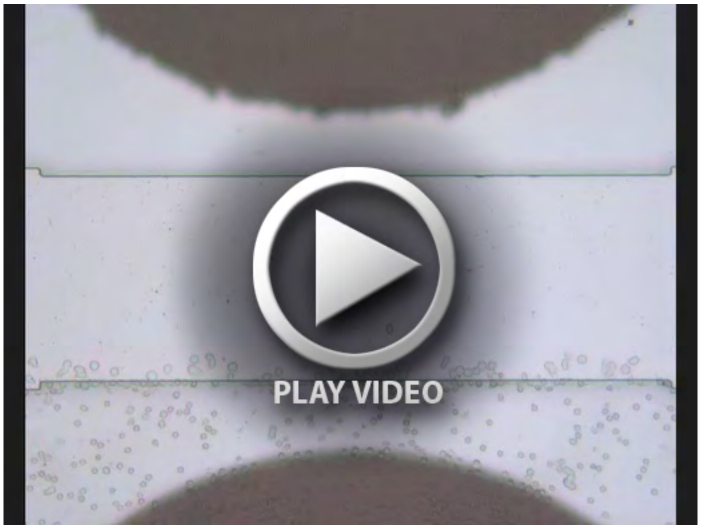

Movie S3. Inhibitory effects of Sterigmatocystin on mouse neutrophil chemotaxis at 100 μM.

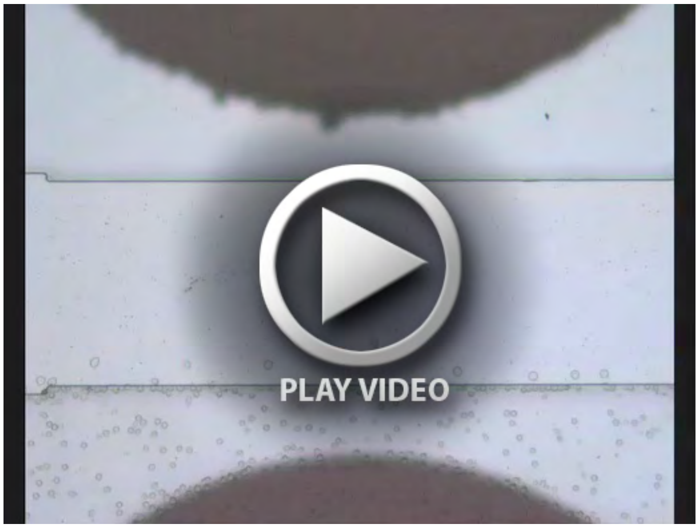

Movie S4. At higher concentration (20 μM), most of PF1052 treated neutrophils remained stationary with a few outliers.

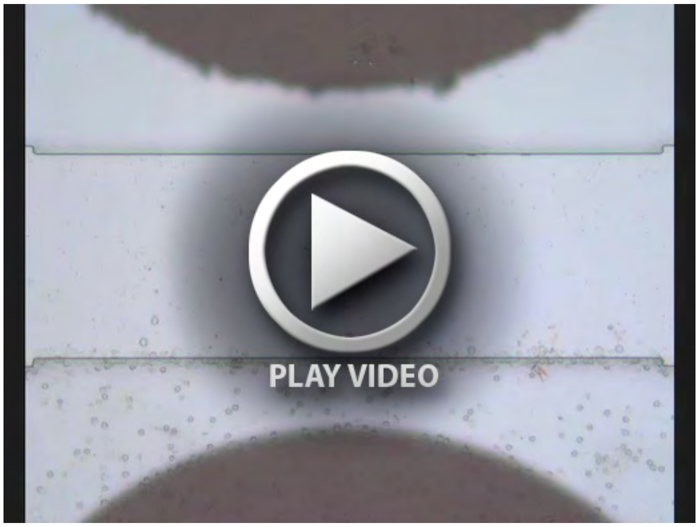

Movie S5. Nearly all neutrophils treated by Sterigmatocystin at 200 μM failed to migrate out.

Supplementary Table S1. Natural product library ID and candidate hits.

Plate ID	Candidate after one round of screening	Confirmed
XF06-1	A10, E07	
XF06-2	A08,A10,H08	A10
XF06-3	D08	
XF06-4	D10	
XF06-5	B03,B05 ,C05	B03
XF06-6	B05,B09	
XF06-7	G04	
XF06-8		
XF06-9		
XP06-1	A10,D01,D02,D03 ,D04 ,D05	
XP06-2	E04,E08,F04 ,F05,F06	
XM01-1	B2,B3,D5,F2,H2,H3,H4,H5	
MB01	B1,B10,C6	

Table S1. Natural product library ID and candidate hits.
